# Microsatellite data suggest significant population structure and differentiation within the malaria vector *Anopheles darlingi *in Central and South America

**DOI:** 10.1186/1472-6785-8-3

**Published:** 2008-03-26

**Authors:** Lisa Mirabello, Joseph H Vineis, Stephen P Yanoviak, Vera M Scarpassa, Marinete M Póvoa, Norma Padilla, Nicole L Achee, Jan E Conn

**Affiliations:** 1 Department of Biomedical Sciences, School of Public Health, State University of New York at Albany, Albany, New York 12222, USA; 2 Molecular Genomics Core Facility, Wadsworth Center, New York State Department of Health, Slingerlands, New York 12159, USA; 3 Florida Medical Entomology Lab, Vero Beach, Florida 32962, USA; 4 Coordenação de Pesquisas em Entomologia, Instituto Nacional de Pesquisas da Amazônia, Av. André Araújo 2936, Manaus, 69011-970, AM, Brasil; 5 Programa de Pesquisas em Malaria, Instituto Evandro Chagas, Br. 316, km 7, s/n, 67.030-000, Ananindeu, Pará, Brasil; 6 Medical Entomology Research and Training Unit Guatemala (MERTU/G), c/o US Embassy, APO Miami, FL 43024, USA; 7 Department of Preventive Medicine and Biometrics, Uniformed Services University of the Health Sciences, Bethesda, MD 20814, USA; 8 Griffin Laboratory, Wadsworth Center, New York State Department of Health, Slingerlands, New York 12159, USA

## Abstract

**Background:**

*Anopheles darlingi *is the most important malaria vector in the Neotropics. An understanding of *A. darlingi*'s population structure and contemporary gene flow patterns is necessary if vector populations are to be successfully controlled. We assessed population genetic structure and levels of differentiation based on 1,376 samples from 31 localities throughout the Peruvian and Brazilian Amazon and Central America using 5–8 microsatellite loci.

**Results:**

We found high levels of polymorphism for all of the Amazonian populations (mean *R*_S _= 7.62, mean *H*_O _= 0.742), and low levels for the Belize and Guatemalan populations (mean *R*_S _= 4.3, mean *H*_O _= 0.457). The Bayesian clustering analysis revealed five population clusters: northeastern Amazonian Brazil, southeastern and central Amazonian Brazil, western and central Amazonian Brazil, Peruvian Amazon, and the Central American populations. Within Central America there was low non-significant differentiation, except for between the populations separated by the Maya Mountains. Within Amazonia there was a moderate level of significant differentiation attributed to isolation by distance. Within Peru there was no significant population structure and low differentiation, and some evidence of a population expansion. The pairwise estimates of genetic differentiation between Central America and Amazonian populations were all very high and highly significant (*F*_ST _= 0.1859 – 0.3901, *P *< 0.05). Both the *D*_A _and *F*_ST _distance-based trees illustrated the main division to be between Central America and Amazonia.

**Conclusion:**

We detected a large amount of population structure in Amazonia, with three population clusters within Brazil and one including the Peru populations. The considerable differences in *N*_e _among the populations may have contributed to the observed genetic differentiation. All of the data suggest that the primary division within *A. darlingi *corresponds to two *white *gene genotypes between Amazonia (genotype 1) and Central America, parts of Colombia and Venezuela (genotype 2), and are in agreement with previously published mitochondrial *COI *gene sequences interpreted as incipient species. Overall, it appears that two main factors have contributed to the genetic differentiation between the population clusters: physical distance between the populations and the differences in effective population sizes among the subpopulations.

## Background

*Anopheles *(*Nyssorhynchus*) *darlingi *is the most efficient malaria vector in the Neotropics, and is responsible for transmission of *Plasmodium falciparum*, *P. vivax *and *P. malariae *[1-5]. Although recently shown to be somewhat of an opportunistic blood-feeder in eastern Amazonian Brazil [6], *A. darlingi *is regarded as the most anthropophilic anopheline in the Americas [7,8]. *Anopheles darlingi*'s range extends from northeastern Argentina to southern Mexico, with a discontinuity in Nicaragua, Costa Rica, and Panama, hypothesized to be the result of an initial colonization event from northern South America into Central America, followed by a modern extinction event in these three countries [9,10]. A complete understanding of *A. darlingi*'s population structure and the processes responsible for the distribution of differentiation is important to vector-based malaria control programs and for identifying heterogeneity in disease transmission as a result of discrete vector populations [11,12]. Susceptibility to *Plasmodium *infection, survival and reproductive rates, degree of anthropophily, and the epidemiology of malaria in the human host may all be affected by genetic variation in vector populations [13,14].

*Anopheles darlingi *exhibits variation in biology [8,15-18], morphology [9,19,20], chromosomes [21,22], isozymes [9,23-25], mtDNA [26-28], Random Amplified Polymorphic DNA (RAPDs) [9], rDNA ITS sequences [9,10,29], and nuclear *white *sequences [10]. The original suggestion that *A. darlingi *is a species complex [17,23] was refuted by Manguin and others [9] with a genetic and morphologic survey, although recent mitochondrial and nuclear range-wide studies provide support for the initial hypothesis [10,28]. Mirabello and Conn [28] found a significant genetic division with mtDNA cytochrome oxidase I (*COI*) data between populations in (1) Amazonia and southern Brazil, and those in (2) Central America and northwestern Colombia. This division was further evaluated with the single copy protein-coding nuclear *white *gene [30,31] and rDNA internal transcribed spacers (ITS) 1 and 2 as independent markers [10]. The *white *gene detected two genotypes, 1 and 2, with significantly different polymorphism statistics (genotype 1 nucleotide diversity = 0.00451; genotype 2 nucleotide diversity = 0.00130), high levels of genetic differentiation (*F*_ST _= 0.7104; Hudson's statistics, *H*_*S*_, = 0.5163, *K*_*S*_, = 0.7161, *Z*_*S*_, = 8.7577, *S*_*nn *_= 0.9253; all *P *< 0.0001), and these genotypes were fairly well-supported monophyletic clades [10]. Together the *white*, ITS, and *COI *gene data confirm a deep divergence between (genotype 1) Amazonia and southern Brazil, and (genotype 2) Central America, Colombia, and Venezuela, and these data are interpreted as incipient speciation within *A. darlingi *[10,28]. The divergence was hypothesized to have occurred during the early to late Pleistocene, most likely shaped by complex Pleistocene climatic changes leading to refugial isolation [10].

Many of the anopheline species responsible for malaria transmission are members of species complexes composed of closely related cryptic species [32]. Most notably, the well-studied African *A. gambiae *complex, which includes seven isomorphic and closely related mosquito species [33], as well as incipient species within *A. gambiae *s.s. [14,34,35]. The members of this complex are highly variable, and also display a large amount of adaptive genetic variation [36]. The recently identified *A. darlingi *incipient species may have differential susceptibility to malaria parasites, and (or) subtle ecological or behavioral differences that could require modifications to vector control efforts. Therefore, a restriction enzyme digestion was designed to distinguish *A. darlingi *genotypes 1 and 2 [Mirabello and others in submission].

Microsatellites are highly polymorphic genetic markers that evolve much faster than mitochondrial or nuclear genes, and are particularly useful for resolving the structure of populations at a finer geographical and evolutionary scale. They have been extensively used for population studies of anophelines, including studies of *A. darlingi *in the Brazilian Amazon [37,38] and between incipient species [14,39-41]. The microsatellite analyses of *A. darlingi *in Amazonian Brazil found that all populations had high genetic variability and departures from Hardy-Weinberg Equilibrium due to heterozygote deficits most likely caused by the Wahlund effect or selection in the east [37] and null alleles in western and central Amazonian populations [38]. There was significant differentiation between *A. darlingi *northeast and southeast of the Amazon River attributed to isolation by distance [37]; central and western Amazonian Brazil populations also demonstrated a correlation between genetic and geographic distance, although, the data supported little genetic structure in this region of the Amazon [38] as compared to that observed in eastern Amazonian Brazil [37].

Here we analyze the molecular variation of *A. darlingi *throughout the Brazilian [combined with previous data from 37,38] and Peruvian Amazon, within Central America in Guatemala and Belize, and between the incipient species described as genotypes 1 and 2 [10] using microsatellite loci. Brazil and Peru have the first and second highest number of reported malaria cases (599,960 cases reported in Brazil; 124,746 in Peru), respectively, in the Neotropics [42] and the majority of cases are reported from the Amazon delta region, attributed to transmission by *A. darlingi*. *Anopheles darlingi *is hypothesized to have been introduced into the Peruvian Amazon in the early 1990's from Brazil [43,44], and in 1997 there was a huge re-emergence of malaria attributed to its presence [45,46].

Within Central America, in Guatemala and Belize, there is a much lower incidence of malaria (33,525 cases reported in Guatemala; 844 in Belize) [42], *Anopheles albimanus *is considered to be the primary malaria vector overall [42], and there is great geographic diversity, in particular there are high mountain ranges (e.g., the Guatemalan Highlands in southern Guatemala and Maya Mountains in western Belize and eastern Guatemala) and lowlands (e.g., Petén lowlands in northern Guatemala and coastal lowlands of Belize) that may restrict gene flow. A more thorough understanding of *A. darlingi*'s population structure and contemporary gene flow patterns in these regions is necessary if vector populations are to be successfully controlled.

We use the variation at 5–8 microsatellite loci [47] from 1,376 *A. darlingi *to address the following questions: Is there population structure within the Peruvian Amazon and throughout Amazonia? Is there a signature of a population expansion around Iquitos, Peru as expected if *A. darlingi *had been recently introduced? Does the level of differentiation observed between genotypes 1 and 2 support the hypothesis of incipient speciation? Is there differentiation within Central America? What are the main forces responsible for the partitioning of genetic variation – geographic distance, physical barriers, or demographic history?

## Methods

### Mosquito collections

Adult *A. darlingi *were field collected outdoors using human landing catches, resting in vegetation near houses, on the outer walls of houses, or at livestock corrals, and identified morphologically [48]. Informed consent was obtained from all collectors, and the Biosafety Committee at the Instituto Evandro Chagas in Belém, state of Pará, Brazil, and New York State Department of Health Institutional Review Board (IRB) reviewed and approved the project. Approval from the IRB board of the Centers for Disease Control and Prevention and the Universidad del Valle de Guatemala was obtained for the collections in Guatemala. The human landing catch protocol used in the Peruvian localities was approved by the US Naval Medical Research Center Detachment in Iquitos, Peru. The mosquito landing catch protocol for Belize was deemed an occupational hazard by Uniformed Services University of the Health Sciences (USUHS) at the time of collections (2006) and appropriate precautions were undertaken. Table 1 lists all the collection and locality information, along with the number of mosquitoes genotyped per site. The collection information for LI, GA, STN, ARA, BEL, MOJ, and PEX is given in Conn *et al*. [37]; for MAC, PVE, SMI, COA, NAI, CAS, PUR, RBR, and BAN in Scarpassa and Conn [38]. All mosquitoes, except those from the collection localities in Scarpassa and Conn [38], are stored at -80°C in the Conn Laboratory at the Wadsworth Center.

### Genotyping and data analysis

Individuals were first determined to be *A. darlingi *genotype 1 or 2 using a *white *gene restriction enzyme digestion [Mirabello and others in submission]. Eight microsatellite loci [47] were amplified and genotyped, as previously described [37], by the New York State Department of Health Wadsworth Center Genotyping Core facility. Data were then analyzed using GENOTYPER 3.7 software (Applied Biosystems, Foster City, CA). Datasets from Conn *et al*. [37] and Scarpassa and Conn [38], consisting of *A. darlingi *samples from eastern, central and western Amazonian Brazil, were included in the analyses and the majority of these samples were re-genotyped using GENOTYPER for consistency of allele size calls.

**Table 1:** *Anopheles darlingi *collection information.

Site # Locality (Abbr.)		Latitude/Longitude Coordinates	*N*	Date	Collector
**Peru**					
1	Zungarococha (ZUN)	3° 49' 33.92" S, 73° 21' 4.72" W	52	1/06	S.P. Yanoviak
2	Padre Cocha (PCO)	3° 42' 12.38" S, 73° 16' 58.48" W	52	1/06	J.E. Ramírez
3	Mazan (MAZ)	3° 29' 32.32" S, 73° 14' 30.52" W	50	2/06	J.E. Ramírez
4	Nauta (NAU)	4° 30' 41.65" S, 73° 35' 8.74" W	53	2/06	C. Valderrama
5	Piura, Rio Tigre (PRT)	4° 6' 35.76" S, 74° 25' 5.16" W	35	2–3/06	C. Valderrama
6	Shishita, Pevas (SHP)	3° 22' 34.61" S, 71° 43' 38.42" W	58	3/06	E. Requena
7	San Esteban (SAE)	3° 56' 48.55" S, 70° 30' 57.56" W	50	3/06	E. Requena
**Brazil**					
8	Boa Vista (BV)	2° 49' N, 60° 40' W	57	7/03	J.E. Conn,, M.M. Póvoa
9	Palito (PLT)	6° 32' 113" S, 55° 78' 817" W	58	7/03	J.E. Conn,, M.M. Póvoa
**Belize**					
10	Caves Branch (CAV)	17° 9.000" N, 88° 40.030" W	45	3/06	N.L. Achee
11	Golden Stream (GOL)	16° 21.814" N, 88° 47.920" W	41	3/06	N.L. Achee
12	Sibun (SIB)	17° 8.887" N, 88° 37.689" W	47	3/06	N.L. Achee
**Guatemala**					
13	San Pablo (SPB)	15° 58' 5.88" N, 90° 47' 24" W	47	9/00	N. Padilla
14	Santa Rosa (SRO)	15° 58' 38.64" N, 90° 50' 53.88" W	49	9/00	N. Padilla
15	El Peñon (ELP)	16° 1' 47.64" N, 90° 46' 29.64" W	47	9/00	N. Padilla

Total individuals newly genotyped = 741: in Peru = 350, Brazil = 115, Belize = 133, Guatemala = 143; *N*, sample size; Date, collection month/year.

The analyses were performed in two ways: 1) including all of the amplified loci from each population (a variation of 5, 7 and 8 loci); and, 2) including only the 5 loci that were amplified from all of the populations (*ADC02, ADC28, ADC110, ADC137*, and *ADC138*).

For each locality, summary polymorphism statistics were generated using Fstat 2.9.3 [49]. Deviations from Hardy-Weinberg equilibrium were assessed per locus and per locality, and linkage disequilibrium between pairs of loci within each locality using Fstat. The significance of these tests was determined using the randomization approach that applies Bonferroni corrections in Fstat. Within each locality the frequency of null alleles was determined using the Brookfield 2 estimate [50], and the allele and genotype frequencies were then adjusted accordingly in MICRO-CHECKER 2.2.3 [51]. The null allele-adjusted dataset was compared to the original dataset to investigate the impact of null alleles on estimations of genetic differentiation.

Genetic differentiation was estimated by calculating *F*_ST _between pairs of populations within and between *A. darlingi *samples using Arlequin 2.001 [52] and GENEPOP 1.2 [53]. The number of migrants per population per generation (*N*_m_) between localities was estimated from pairwise *F*_ST _[54]. An analysis of molecular variance (AMOVA) was used to examine the distribution of genetic variation in Arlequin using *F*_ST_. We focused on estimates of *F*_ST _performed under the infinite alleles model (IAM) because this model is considered more reliable when fewer than 20 microsatellites are used [55]. The significance for all calculations was assessed by 10,000 permutations and the *P*-values were Bonferroni adjusted. The isolation by distance model was investigated as a potential explanation for the observed population differentiation. The significance of the regression of genetic distance on geographic distance between sample pairs was tested using a Mantel test [56] with 10,000 permutations using Arlequin.

Several approaches were used to investigate the relationships among populations. We constructed a neighbor-joining (NJ) tree based on pairwise Nei *et al*.'s [57]*D*_A _distance and *F*_ST _values for all the populations using MEGA version 3.1 [58]. A Bayesian clustering analysis was implemented in STRUCTURE 2.1 [59,60] with a burn-in period of 500,000 chains and 1,000,000 Markov chains Monte Carlo replications for each of *K *= 1 to 8. This clustering method estimates the most probable number of discrete populations with no *a priori *assumptions of population structure. Each simulation was done in triplicate to assess the consistency of the data.

Inferences of non-neutral evolution were investigated using two tests, the homozygosity test implemented in BOTTLENECK 1.2.02 [61], and Kimmel's β-imbalance index [62] using the β_1 _estimator [63]. Significance of the homozygosity test was evaluated by simulations implemented in BOTTLENECK. The homozygosity test was performed under the step-wise mutation model (SMM) and the two-phase mutation model (TPM) with one-step mutations occurring at a frequency of 90% of the total. SMM and TPM (specifically the 90% model) are considered the more realistic microsatellite mutation models [64], thus only these results are given. The β-imbalance index, as well as 95% confidence intervals, were estimated using a SAS program written and run by T. Lehmann [65]. The long-term effective population size (*N*_e_) was estimated using NeEstimator version 1.3 [66] based on the linkage disequilibrium and heterozygote excess models.

## Results

All individuals collected from Peru and Brazil were classified as *white *genotype 1 and all those collected from Guatemala and Belize were classified as *white *genotype 2 [10, Mirabello and others in submission].

### Genetic diversity

From the original set of eight microsatellite loci designed for *A. darlingi *from eastern Amazonian Brazil [47], one locus (*ADC107*) did not amplify in any of the individuals collected from Peru and three loci (*ADC01*, *ADC29*, and *ADC107*) did not amplify in any of the individuals from Belize or Guatemala. Seven loci were genotyped from 350 individuals in Peru, five loci from 276 individuals from Central America (143 from Guatemala and 133 from Belize), and all eight loci from 57 individuals from BV and 58 from PLT, Brazil (Table 1). In combination with previously amplified specimens from eastern Amazonian Brazil (*N *= 254) [37] and from central and western Amazonian Brazil (*N *= 381) [38], the total dataset consisted of 1,376 *A. darlingi*. The levels of polymorphism were high in Amazonian Brazil and Peru, and low in Central America, based on the 5 shared loci: in Peru, the number of alleles per locus ranged from 5–11 and the observed heterozygosity from 0.44 – 0.86 (mean *R*_S _= 7.2, mean *H*_O _= 0.716); in Brazil, the number of alleles per locus ranged from 6–22 and the observed heterozygosity from 0.74 – 0.93 (mean *R*_S _= 8.95, mean *H*_O _= 0.836) [37,38]; and, in Belize and Guatemala, the number of alleles per locus ranged from 2–7 and the observed heterozygosity from 0.12 – 0.68 (mean *R*_S _= 4.3, mean *H*_O _= 0.457) (Additional File 1).

Deviations from Hardy-Weinberg equilibrium, detected by the inbreeding coefficient *F*_IS_, within some samples from Peru (6 of 49 tests), Brazil (BV: 1 of 8 tests, and PLT: 5 of 8 tests), and Central America (4 of 30 tests) indicated heterozygote deficits (Additional File 1). The heterozygote deficits are most likely due to null alleles as they were not genome or population wide and no significant linkage disequilibrium was detected, consistent with the presence of null alleles detected in western and central Amazonian Brazil for *A. darlingi *[38]. In eastern Amazonian Brazil, heterozygote deficits were associated with linkage disequilibrium most likely due to either selection or the Wahlund effect (population substructure) [37]. To determine if the null alleles impacted our population genetic analyses, we performed these analyses both before and after the dataset was adjusted for estimated null allele frequencies. The effect of this treatment was minimal and did not significantly change the degree or statistical significance of the estimated parameters.

### Population structure and differentiation

For analyses of population structure and differentiation, only the data based on the five loci that were amplified from the entire sample set are shown.

**Figure 1:**
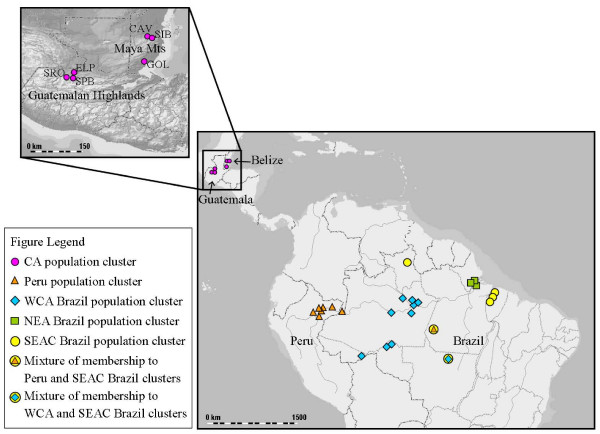
Map of collection sites and population clusters. The inset illustrates the geography (mountains are represented by lighter areas) and location of the sites in Guatemala and Belize. Each symbol corresponds to a collection site and membership to one or a mixture of the five population clusters, outlined in the figure legend; percent membership below 15% is not shown, only mentioned in the text. CA, Central America; WCA, western and central Amazonian Brazil; NEA, northeastern Amazonian Brazil; SEAC, southeastern Amazonian and central Brazil.

An unsupervised Bayesian clustering analysis revealed five population clusters. *Anopheles darlingi *seemed to cluster mostly on the basis of physical proximity of sampling sites, with four population clusters within Amazonia and one cluster including all specimens from Belize and Guatemala (Figure 1). The five population clusters had the following proportion of specimen membership from each collection site: (1) 95.5% of the individuals from Belize (CAV, GOL, and SIB) and Guatemala (SPB, SRO, and ELP); (2) 95.8% of the individuals from Peru (ZUN, PCO, MAZ, NAU, PRT, SHP, and SAE), and 26.8% from PLT, Brazil; (3) 94.3% of the individuals from western and central Amazonian (WCA) Brazil (MAC, PVE, SMI, COA, NAI, CAS, PUR, RBR, and BAN), 23.6% from PEX, and 14.1% from PLT, Brazil; (4) 96.5% of the individuals from northeastern Amazonian (NEA) Brazil (LI, GA, and STN), and11.2% from PEX, Brazil; (5) 96.9% of the individuals from southeastern Amazonian Brazil (ARA, BEL, and MOJ), 92.6% from BV, 50.3% from PEX, and 40.8% from PLT, Brazil. Allele frequency distributions among the five population clusters showed that there is a mix of shared and private alleles, and particularly more private alleles in the Central America cluster (data not shown). The five clusters show differences in the most common allele size and allele distributions for each locus, most markedly between Central America and the Amazonian populations.

**Figure 2:**
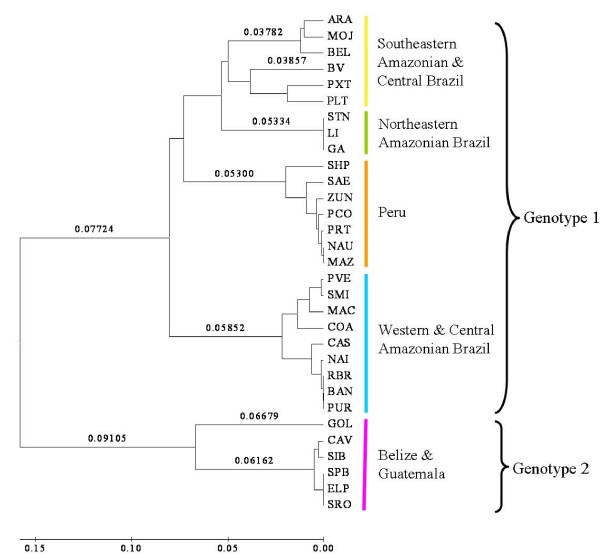
*F*_ST _distance-based NJ tree of the pairwise comparisons among all populations. The mean pairwise *F*_ST _values are proportional to the branch lengths (see scale bar). The pairwise estimates of *F*_ST _were 100% significantly different (*P *< 0.05 after sequential Bonferroni correction) when samples from the two genotypes and among the five population clusters were compared.

Both the *D*_A _and *F*_ST _distance-based trees illustrated two main population clusters: one including all of the samples from Belize and Guatemala, and the other including all samples from Amazonian Peru and Brazil (Figure 2). These two clusters are consistent with genotypes 1 and 2 [10]. Within the Amazonian cluster there were four smaller subclusters, corresponding to those detected with the Bayesian analysis (2–5 above).

An AMOVA using the five population clusters detected with the Bayesian analysis as the groupings, found that 18.9% of the variance was explained at the among groups level, and 77.9% at the within populations level. In an AMOVA using the two genotypes, Amazonia and Central America, as the groupings, 20.1% of the total variance was explained at the between groups level and 70.1% within populations. All of the global *F*_ST _estimates revealed significant overall genetic structure (*P *< 0.001). The majority of the genetic diversity in *A. darlingi *is accounted for by within-population differences among individuals.

Within genotype and population cluster levels of differentiation ranged from low to moderate. Within Central America (genotype 2) there was mostly low non-significant differentiation (mean *F*_ST _= 0.047, 33.3% *P *< 0.05), except for between GOL and all other subpopulations there was a moderate amount of significant differentiation (*F*_ST _range of 0.1063–0.1489) (Table 2). Within Amazonia (genotype 1) there was a moderate level of significant differentiation (mean *F*_ST _= 0.1244, 89% *P *< 0.05), and within the four Amazonian population clusters the mean *F*_ST _ranged from -0.002 to 0.082 (70.6% *P *< 0.05) (Table 2). Pairwise *F*_ST_comparisons among all of the samples ranged from -0.0005 to 0.3901, with the largest values corresponding to comparisons between genotypes 1 and 2 (0.1859 – 0.3901). The mean pairwise estimates of genetic differentiation between Central America and the four population clusters in Amazonia were high (*F*_ST _= 0.2161 – 0.3625) (Table 3). Comparisons between the population clusters within Amazonia revealed moderate differentiation among the Amazonian clusters (mean *F*_ST _= 0.0751 – 0.1813), particularly between Peru and WCA Brazil, Peru and NEA Brazil, and NEA and WCA Brazil (Table 3). All of the comparisons between genotypes 1 and 2, and between the five population clusters, revealed significant differentiation (*P *< 0.05) after correction for multiple tests. Estimates of gene flow revealed little or no recurrent gene flow between genotypes 1 and 2, and reduced gene flow among the population clusters in Amazonia (Table 3). Within each population cluster, there was moderate to high levels of gene flow, particularly high within NEA Brazil (Table 3).

**Table 2:** Pairwise genetic differentiation within populations of *A. darlingi *based on the 5 shared loci.

	CA	Peru	WCA Brazil^†^	NEA Brazil^‡^	SEAC Brazil^‡^	Amazonia
*N*	276 (6)	350 (7)	381 (9)	122 (3)	247 (6)	1100 (25)
Mean *F*_ST_	0.0470	0.0185	0.0292	-0.0016	0.0823	0.1244
Range	-0.009–0.164	-0.0006–0.061	-0.0002–0.074	-0.001–0.003	0.023–0.160	-0.002–0.220
Significance	5/15	11/21	27/36	0/3	15/15	267/300

CA includes CAV, GOL, SIB, SPB, SRO, and ELP; Peru includes ZUN, PCO, MAZ, NAU, PRT, SHP, and SAE; WCA Brazil includes MAC, PVE, SMI, COA, NAI, CAS, PUR, RBR, and BAN; NEA Brazil includes LI, GA, and STN; SEAC Brazil includes ARA, BEL, MOJ, PEX, BV, and PLT; Amazonia, includes all populations in Peru and Brazil; ^†^, [38]; ^‡^, some samples are from [37]; *N*, number of individuals (number of localities); significance = *P *< 0.05 after sequential Bonferroni correction.

**Table 3:** Pairwise estimates of genetic differentiation (*F*_ST_) below the diagonal (average distance in km) and gene flow (*N*_M_) above the diagonal among *A. darlingi *populations, and within population gene flow along the diagonal.

	CA	Peru	WCA Brazil^†^	NEA Brazil^‡^	SEAC Brazil^‡^
CA	5.07	0.43	0.59	0.50	0.90
Peru	0.3625* (2923)	13.26	1.36	1.43	2.36
WCA Brazil^†^	0.2963* (3883)	0.1556* (1249)	8.31	1.13	2.34
NEA Brazil^‡^	0.3326* (4609)	0.1484* (2451)	0.1813* (1370)	156.5	3.08
SEAC Brazil^‡^	0.2161* (4593)	0.0959* (2270)	0.0967* (1243)	0.0751* (673)	2.79

See Table 2 for abbreviations; ^†^, samples from [38]; ^‡^, some samples are from [37]; *, *P *< 0.05 after sequential Bonferroni correction.

The significant differentiation between genotypes 1 (the 4 population clusters in Amazonian Peru and Brazil) and 2 (Central America) was genomewide, as shown by independent analyses for each of the five loci revealing significant differentiation between them (data not shown). The differentiation between the genotypes varied in magnitude, with the highest level of differentiation observed at locus *ADC28 *and the lowest at locus *ADC110*. The differentiation among the four Amazonian population clusters was not genomewide, as shown by independent analyses of the eight loci (data not shown). Loci *ADC137 *and *ADC01 *(only amplified in the Amazonian populations) did not show significant differentiation among the Amazonian population clusters. The highest level of differentiation among all of the Amazonian population clusters was at locus *ADC02*.

**Figure 3:**
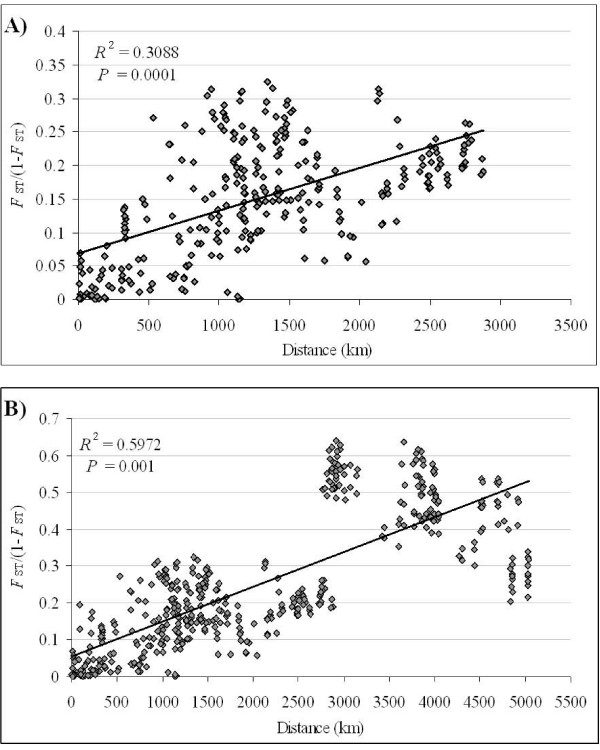
Scatterplot of pairwise *F*_ST _values against geographic distance separating pairs of localities. A) shows the plot for Amazonian subpopulation comparisons; B) shows the plot for all subpopulation comparisons. The regression line is shown through the points.

Tests of isolation by distance were performed separately for all of the populations together, for each population cluster, and for all of Amazonia. For all of the populations together (4–5028 km) and all of Amazonia (4–2878 km), there was a significant positive correlation between geographic distance and genetic differentiation based on the Mantel test (All: *R*^2 ^= 0.5972, *P *= 0.001; Amazonia: *R*^2 ^= 0.3088, *P *= 0.0001) (Figure 3). The data did not fit the isolation-by-distance model within Peru (16–433 km), NEA Brazil (4–8 km), or Central America (4–270 km). Within WCA Brazil [38] and the southeastern Amazonian and central (SEAC) Brazil (ARA, BEL, MOJ, PEX, BV, and PLT) (10–1601 km) population cluster (*R*^2 ^= 0.6053, *P *= 0.007), there was a significant positive association between distance and *F*_ST_. The results suggest that the genetic differentiation observed between *A. darlingi *populations is primarily due to restricted gene flow by geographic distance. Although, between Peru populations and those in NEA Brazil and SEAC Brazil the average distance is 2451 km and 2270 km and the mean pairwise *F*_ST _is 0.1484 and 0.0959, respectively; and, the average distance between Peru and Central American populations is 2923 km and the mean pairwise *F*_ST _is 0.3625, which demonstrates that the large genetic differentiation is not accompanied by correspondingly large difference in geographic distance.

### Demographic inference

Statistics designed to detect a population expansion were calculated. These tests are based on the premise that an expanded population mutations are more likely to be recent and, therefore, would only differ in size by a single microsatellite repeat unit. The homozygosity test contrasts the homozygosity or expected heterozygosity estimated based on allele frequencies with that estimated based on the number of alleles and sample size [61]. The β-imbalance index is based on the imbalance between the variance in allele size and heterozygosity at a locus [62]. These statistics are expected to be equal in a neutral locus at mutation-drift equilibrium (MDE). The majority of the populations did not significantly depart from MDE. Many of the significant homozygosity test results were dependent on the mutation model used (Table 4). The homozygosity test detected significant departures from equilibrium across many loci within Peru (MAZ, NAU, and SAE), WCA Brazil (MAC, CAS, NAI, and RBR), in BV and PLT, Brazil, and within Central America (GOL and SPB). The significantly higher heterozygosity based on the number of alleles suggests a recent expansion of these populations. Alternatively, these significant results could be due to a recent influx of rare alleles from genetically distinct populations [61].

**Table 4:** Tests of neutrality for each population.

	Homozygosity test		β-imbalance index
		
	SMM	TPM-90%	
**CA**			
CAV	1/5	0/5	0.97
GOL	1/5*	0/5	1.17
SIB	1/5	1/5	1.43
SPB	2/5*	2/5*	2.78
SRO	2/5	1/5	1.21
ELP	2/5	1/5	1.51
**Peru**			
ZUN	2/7	3/7	6.75
PCO	3/7	3/7	6.20
MAZ	3/7*	2/7	8.22
NAU	4/7*	2/7	12.69
PRT	3/7	2/7	5.47
SHP	0/7	1/7	3.93
SAE	4/7*	2/7	5.57
**WCA Brazil**			
MAC	2/7**	1/7	4.49
PVE	2/7	3/7	2.61
SMI	2/7	1/7	2.92
COA	2/7	0/7	4.77
NAI	2/7**	1/7**	4.96
CAS	2/7*	0/7	4.82
PUR	2/7	0/7	2.53
RBR	4/7*	3/7	7.14
BAN	4/7	3/7	5.29
**NEA Brazil^‡^**			
LI	--	--	3.68
STN	--	--	4.01
GA	--	--	4.91
**SEAC Brazil^‡^**			
ARA	--	--	1.90
BEL	--	--	5.32
MOJ	--	--	1.49
PEX	--	--	1.24
BV	2/8**	1/8	4.98
PLT	2/8**	1/8	1.85

CA, Central America; ^‡^, the Homozygosity test results for LI, GA, STN, ARA, BEL, MOJ, and PEX are given in [37]; *, all loci together *P *< 0.05; **, all loci together *P *< 0.01; --, no data.

The imbalance index is expected to depart from 1 after a demographic change. Specifically, the imbalance index is expected to be less than 1 after a population expansion and greater than 1 in a population that has expanded after a bottleneck [62]. However, the imbalance index may also be greater than 1 in a population that has experienced a severe bottleneck after an expansion [65]. Although none of these results were statistically significant, the values greater than 1 in Peru, WCA Brazil, NEA Brazil, SPB, Guatemala, BEL and BV, Brazil suggest that in these populations there could be a slight signal of an expansion following a bottleneck or a bottleneck after an expansion.

### Effective population size

The *N*_e _estimates varied considerably among the subpopulations and population clusters, and depending on the model used (Table 5). Under the heterozygote excess model all of the *N*_e _estimates were infinity for both treatment methods (based on 5 vs. 5–8 loci). Under the linkage disequilibrium, the highest overall effective population size estimates for the five population clusters were observed in Central America (*N*_e _= 8, 95% CI: 649.7 – 8) and NEA Brazil (*N*_e _= 8, 95% CI: 2698.2 – 8), based on the 5 common loci. The overall *N*_e _estimate for Peru was high based on 5 loci (*N*_e _= 1161.1, 95% CI: 599.9 – 6815.7), and more moderate based on all 7 amplified loci (*N*_e _= 379, 95% CI: 316.5 – 464.8), which demonstrates a discrepancy in the calculation based on 5 vs. 7 loci. The *N*_e _estimates for BV and PLT, Brazil (Table 5) were similar to the values for WCA Brazil and eastern Amazonian Brazil given in Scarpassa and Conn [38] and Conn and others [37], respectively. In Peru, the highest *N*_e _values were in NAU, SAE, and ZUN, and the lowest in PRT and MAZ. In Central America, the highest *N*_e _values were in GOL, Belize, and SRO, Guatemala, and the lowest in ELP, Guatemala. Overall, within the Central American populations the majority of the effective population sizes were lower than those observed within the Amazon.

**Table 5:** Estimated *N*_e _(columns 1 and 3) based on the linkage disequilibrium (LD) model.

	LD 95% CI (columns 2 and 4)			
	
	Based on 5 loci		Based on 7–8 loci	
Peru	1161.1	599.9 – 6815.7	379	316.5 – 464.8
NAU	8	171.5 – 8	1786	205.6 – 8
PRT	93.6	41.3 – 8	95.2	55.8 – 258.7
MAZ	131.8	61.2 – 4139.3	61.7	46.4 – 87.9
SHP	177.2	78.3 – 8	159	84.3 – 228.7
SAE	1044.9	139.5 – 8	1929	245.3 – 8
PCO	220.2	72.8 – 8	453.7	148.1 – 8
ZUN	8	3405.4 – 8	8	251.0 – 8
CA	8	649.7 – 8	--	--
Belize	216	96.3 – 8274.7	--	--
CAV	70.5	29 – 8	--	--
GOL	526.6	45.9 – 8	--	--
SIB	84.7	30 – 8	--	--
Guatemala	773.8	163.9 – 8	--	--
SPB	62.6	27.9 – 1265.4	--	--
SRO	8	72.6 – 8	--	--
ELP	35.8	17.8 – 129.3	--	--
WCA Brazil^†^	233.6	195.4 – 284.0	202.4	186.1 – 220.8
NEA Brazil^‡^	8	2698.2 – 8	1405.8	539.0 – 8
SEAC Brazil^‡^	90.2	81.4 – 100.2	101.4	96.3 – 106.9
BV	413.1	114.6 – 8	324.7	155.0 – 8
PLT	144.2	90.4 – 316.8	120.7	96.50 – 158.9

See Table 2 for abbreviations; ^†^, the results for MAC, PVE, SMI, COA, NAI, CAS, PUR, RBR, and BAN are given in Scarpassa and Conn [38]; ^‡^, the results for LI, GA, STN, ARA, BEL, MOJ, and PEX are given in [37]; CI, confidence intervals; 8, infinity; --, no data.

## Discussion

The microsatellites used in this study are highly polymorphic, and thus are useful for exploring *A. darlingi*'s population genetic structure. *Anopheles darlingi *is a species characterized by moderate levels of molecular variability [21-29,67], and our microsatellite analysis is in agreement with earlier studies. The high allelic diversity and heterozygosity observed in Peru, BV and PLT, Brazil are similar to the results of previously analyzed Amazonian Brazil *A. darlingi *[37,38], *A. albimanus *[68] in Latin America, and African vectors *A. gambiae *[69] and *A. funestus *[40,41]. We detected significant departures from HW equilibrium due to heterozygote deficits, and no linkage disequilibrium, in loci in Peru, BV and PLT in Brazil, and in Central America. In contrast, most loci across all populations in eastern Amazonian Brazil had deficits with linkage disequilibrium, interpreted as due to either the Wahlund effect or selection [37]. In western and central Amazonian Brazil significant deficits were detected in 50.79% of the HW equilibrium tests, with only minimal linkage disequilibrium interpreted as null alleles [38]. An allozyme study of two Amazonian populations detected significant deviations from HW equilibrium in 7/8 loci examined [70], although no significant deviations were detected in earlier allozyme studies [9,24]. The high levels of heterozygote deficits and null alleles could be the result of an accumulation of mutations in the primer binding sites which may be a consequence of the microsatellite library being constructed of *A. darlingi *from eastern Amazonian Brazil [47]. The incidence of null alleles found in *A. darlingi *is similar to that reported from many anopheline microsatellite studies [12,35,39-41]; perhaps mosquitoes with large population sizes and high levels of polymorphism are more likely to have null alleles [39].

The eight microsatellite loci used in this study have not been physically mapped to *A. darlingi *polytene chromosomes. Therefore, their location with respect to polymorphic chromosome inversions is unknown, and such information may modify the interpretation of the data because neutrality cannot be assumed. Since the analyses were done in two ways, including all amplified loci (5–8) and including only the 5 loci amplified from all populations, we were able to compare the results of these two treatment methods. The differentiation and mean heterozygosity (Table 2) results were not significantly different between these two methods; both recovered very similar values. The allelic richness (Table 2) and the neutrality test estimates showed a little more variance, and the effective population size estimates a large disparity between the two treatment methods, which demonstrates that this test is more sensitive and should be interpreted with caution. Although there was variance in these estimates, the same trends were shown in both treatments.

Substantial population structure was found in Amazonia, which was undetected with more conservative nuclear markers and isozymes [9,10,24,71]. Four population clusters were detected in Amazonia, three in the Brazilian Amazon (northeastern Amazonia, southeastern Amazonia and central, and western and central Amazonia) and one including the Peruvian Amazon subpopulations, attributed to an isolation-by-distance effect. There was a moderate amount of significant differentiation and reduced gene flow between these Amazonian population clusters. The considerable differences in *N*_e _among the populations may have contributed to the observed genetic differentiation [72,73]. The level of differentiation among the Amazonian population clusters is comparable to that detected between *A. albimanus *populations from Central and South America (*F*_ST _= 0.114) [68], among *A. gambiae *populations in west Africa separated by > 200 km (*F*_ST _= 0.034–0.167) [74] and those separated by the Great Rift Valley complex in Kenya (*F*_ST _= 0.104) [75], as well as between *A. funestus *populations from west, central, and eastern Africa (*F*_ST _= 0.110) [11]. An earlier mtDNA study of *A. darlingi *[28], although lacking western Amazonian Brazil samples, detected considerable population structure throughout South America that is congruent with some of the Amazonian differentiation detected here; specifically, differentiation across the Amazon River [37] and between the NEA and SEAC Brazil population clusters was also detected with mtDNA [28]. The main forces responsible for partitioning the genetic variation in Amazonian are most likely the result of geographic distance and/or differing demographic histories, rather than physical barriers (*e.g*., rivers or mountains).

Within the WCA Brazil population cluster there was little genetic structure and differentiation, and the isolation-by-distance model explained nearly all of the differentiation observed [38]. Within the NEA Brazil population cluster there was no significant population structure or differentiation, likely because these three localities are 4–8 km apart and probably a single population. Within the SEAC Brazil cluster there was more structure and significant differentiation than observed for the other Amazonian clusters, which is explained by isolation-by-distance and also may be affected by the differing effective population sizes among these subpopulations. The two central Amazonian Brazil populations, PEX and PLT, were an admixture of the Amazonian clusters. PEX was primarily an admixture of SEAC and WCA Brazil populations, which are the two nearest population clusters. Interestingly, PLT shared identity primarily with the SEAC populations, which are in close proximity (although not the closest), and secondly shared identity with the Peruvian populations that are 1611–2044 km apart. BV, the northern Amazonian Brazil locality, was most similar to the southeastern Amazonian Brazil populations, which again were not the nearest. This demonstrates that their population identity was not solely based on proximity, and may be influenced by demographic history, migration, and/or ecology.

Within Peru there was no significant population structure and low differentiation among the seven subpopulations, in agreement with an earlier RAPD-PCR analysis of *A. darlingi *in the Peruvian Amazon that detected high homogeneity among populations (within 60 km) irrespective of different habitat types [76]. We detected little differentiation between the subpopulations even at distances up to 433 km and there was no indication of isolation-by-distance. Most of the significant low differentiation among the subpopulations occurred between samples greater than 120 km apart, except for between PCO-NAU (59 km apart, significant differentiation), PCO-PRT (134 km apart, no significant differentiation), and MAZ-PRT (147 km apart, no significant differentiation). There was a large amount of variability in *N*_e _among the Peru subpopulations (93.6 – 8) that may contribute to the small significant differentiation observed among many of the localities. *Anopheles darlingi *appears to be panmictic in this region of Peru. There is some evidence of a population expansion in MAZ, NAU, and SAE. The expansion in NAU and SAE is reflected in a very large *N*_e _in these localities. Prior to 1991, *A. darlingi *had not been reported around Iquitos, the major Peruvian Amazon city [43,44]. This expansion may reflect the introduction of *A. darlingi *into the Peruvian Amazon possibly from PLT, Brazil, where there is the most genetic similarity, in the early 1990's and the resultant increase in malaria [45,46].

Within Central America there was much less variation (mean *R*_S _= 4.3, mean *H*_O _= 0.457) as compared to within Amazonia (mean *R*_S _= 7.62, mean *H*_O _= 0.742), and there was no evidence of isolation-by-distance. Low haplotype and nucleotide diversity was also observed within Central America with mtDNA *COI *sequences as compared to within South America [28]. The low diversity can be at least partially explained by low effective population sizes in this region, or perhaps these populations suffered a recent population bottleneck due to an unknown historical event. A founder effect resulting from the establishment of the Central American *A. darlingi *populations from only a few individuals from the Colombian population is also consistent with the data [10]. The only significant differentiation observed among the six Central American subpopulations was between GOL, Belize and all other localities (*F*_ST _range of 0.1063–0.1489, all *P *values < 0.05), GOL is separated from the other subpopulations by the Guatemalan Highlands and the Maya Mountains (Figure 1), which may act as a natural barrier, restricting gene flow. There was no significant differentiation between the northern Belize populations (CAV and SIB) and the Guatemalan populations, although they are separated by 257–270 km and by the mountain ranges as well. Therefore, in northern Belize and within Guatemala *A. darlingi *appears to be one panmictic unit. In comparison, *A. albimanus *populations throughout Central America displayed only minor genetic differences using microsatellites, there was weak isolation by distance, throughout Guatemala populations were genetically homogenous between Atlantic and Pacific regions and thus the Guatemalan Highlands did not appear to restrict gene flow [68]. The level of differentiation observed between GOL and the other *A. darlingi *Central American populations was similar to that observed between *A. albimanus *populations in Central and South America [68].

The data suggest that the main division within *A. darlingi *corresponds to Amazonia (genotype 1) and Central America (genotype 2) [10]. Earlier nuclear *white*, ribosomal ITS, and mitochondrial *COI *sequence data together established a deep divergence between genotypes 1 and 2 [10,28], interpreted as incipient species [10]. In the present study, there is marked differentiation between Central America and all four Amazonian population clusters. All pairs of genotype 1 and 2 populations showed a large amount of highly significant differentiation, there was little or no recurrent gene flow between them, they demonstrate different microsatellite allele frequencies and variation, and appear as separate clusters with the Bayesian analysis. The NJ trees based on genetic differentiation and distance both cluster the populations according to the two genotypes. The mixture of shared and private alleles in the Central America population cluster is consistent with shared ancestral polymorphism and a recent divergence between these two genotypes. The presence of a large amount of private alleles suggests some degree of independence between the gene pools [77]. The independent pairwise differentiation analyses of each locus found significant differentiation across the genome between genotypes 1 and 2. The differentiation observed between the genotypes was attributed to isolation by distance, although, as the graph shows (top right portion of Figure 3), the comparisons between Central and South American populations do not fit the positive correlation trend line, and may be a consequence of comparing diverse genetic groups that are geographically separated [11,68]. The level of differentiation observed between genotype 1 and 2 populations was similar to that observed among the closely related *A. dirus*, *A. scanloni*, and *A. baimaii *(former *A. dirus *species A, C, and D, respectively) in Thailand (mean *F*_ST _= 0.263) [39], *A. gambiae *M and S forms (*F*_ST _= 0.1–0.3 [12]; mean *F*_ST _= 0.203 [35]), and between *A. gambiae *and *A. arabiensis *(*F*_ST _= 0.12–0.27 [78]; mean *F*_ST _= 0.349 [35]) using microsatellites. These microsatellite data are consistent with and substantiate the hypothesis, initially proposed based on mitochondrial and nuclear data [10,28], that genotypes 1 and 2 represent incipient species within *A. darlingi*. The divergence between these genotypes was estimated to have occurred during the Pleistocene using mitochondrial data, most likely attributed to complex Pleistocene climatic changes [28].

With the detection of a recent population expansion or the departure from MDE in many of the populations in Amazonia and two populations in Central America, the *F*_ST _values do not translate into meaningful rates of gene flow [79]. In the expanded populations, the migration rates will be overestimated by *F*_ST_, and the differentiation will be underestimated as compared to neutral equilibrium values. Therefore, the low level of differentiation measured within Peru and WCA Brazil may be an underestimation as well as an overestimation of gene flow; and, the differentiation and gene flow between the genotypes and population clusters may be underestimating the current degree of isolation. Despite possible departures from MDE, our large sample sizes and number of populations add statistical power to our study.

## Conclusion

Overall, there was a large amount of population structure in Amazonia, and a primary division within *A. darlingi *between Amazonia (genotype 1) and Central America, parts of Colombia and Venezuela (genotype 2). It appears that two main factors have contributed to the genetic differentiation between the population clusters: physical distance between the populations and the differences in effective population sizes among the subpopulations. Knowledge of *A. darlingi*'s population genetic structure is essential to an understanding of malaria epidemiology and for the success of potential genetic control strategies (release of transgenic mosquitoes refractory to *Plasmodium *infection) that will rely on the ability to target all populations and will require a thorough understanding of the forces that produce and maintain the population structure, especially gene flow [80]. Control strategies involving insecticides will also benefit from knowledge of gene flow, which would allow predictions about the spread of genes conferring insecticide resistance or susceptibility within and between vector populations. These control strategies should take *A. darlingi*'s population structure, and specifically these genotypes, into account.

## Authors' contributions

LM designed the study, extracted and prepared the *A. darlingi *specimens, carried out the analyses, and drafted the manuscript. JHV did most of the PCR and genotyping at the New York State Department of Health Wadsworth Center Genotyping Core facility, assisted with the structure analysis, and gave general microsatellite analysis advice. SPY managed all of the *A. darlingi *collections in Peru. MMP together with JEC provided samples from PLT, BV and those from eastern Amazonian Brazil [37]. VMS provided specimens from western and central Amazonian Brazil [38]. NP provided all of the samples of *A. darlingi *from Guatemala. NLA provided all of the *A. darlingi *samples from Belize. JEC participated in the study design, coordinated collaborations and *A. darlingi *collections, and helped revise the manuscript. All authors read and approved the final manuscript.

## Supplementary Material

Additional File 1 Summary of microsatellite variation at 5–8 loci for *A. darlingi *in Central America, Peru and Brazil. The data provided represent the number of alleles, heterozygosity and inbreeding coeffient values of each microsatellite locus at each locality. Click here for file

## References

[B1] Deane LM (1947). Observações sobre a malária na Amazônia brasileir. Rev Serv Esp Saúde Públ.

[B2] Deane LM (1988). Malaria studies and control in Brazil. Am J Trop Med Hyg.

[B3] de Arruda M, Carvalho MB, Nussenzweig RS, Mararic M, Ferreira AW, Cochrane AH (1986). Potential vectors of malaria and their different susceptibility to *Plasmodium falciparum *and *Plasmodium vivax *in northern Brazil identified by immunoassay. Am J Trop Med Hyg.

[B4] Flores-Mendoza C, Fernandez R, Escobedo-Vargas KS, Vela-Perez Q, Schoeler GB (2004). Natural Plasmodium infections in *Anopheles darlingi *and *Anopheles benarrochi *(Diptera: Culicidae) from eastern Peru. J Med Entomol.

[B5] Grieco JP, Achee NL, Roberts DR, Andre RG (2005). Comparative susceptibility of three species of *Anopheles *from Belize, Central America, to *Plasmodium falciparum *(NF-54). J Am Mosq Control Assoc.

[B6] Zimmerman RH, Galardo AKR, Lounibos LP, Arruda M, Wirtz R (2006). Bloodmeal hosts of *Anopheles *species (Diptera: Culicidae) in a malaria-endemic area of the Brazilian Amazon. J Med Entomol.

[B7] Charlwood JD, Alecrim WA (1989). Capture-recapture studies with the South American malaria vector *Anopheles darlingi*, Root. Ann Trop Med Parasitol.

[B8] Lourenço-de-Oliveira R, Guimarães AEG, Arie M, Fernandes da Silva T, Castro MG, Motta MA, Deane LM (1989). Anopheline species, some of their habits and relation to malaria in endemic areas of Rondonia State, Amazon region of Brazil. Mem Inst Oswaldo Cruz.

[B9] Manguin S, Wilkerson RC, Conn JE, Rubio-Palis Y, Donoff-Burg JA, Roberts DR (1999). Population structure of the primary malaria vector in South America, *Anopheles darlingi*, using isozyme, random amplified polymorphic DNA, internal transcribed spacer 2, and morphologic markers. Am J Trop Med Hyg.

[B10] Mirabello L (2007). Molecular population genetics of the malaria vector *Anopheles darlingi *throughout Central and South America using mitochondrial, nuclear, and microsatellite markers. PhD thesis.

[B11] Michel AP, Ingrasci MJ, Schemerhorn BJ, Kern M, Goff GLE, Coetzee M, Elissa N, Fontenille D, Vulule J, Lehmann T, Sagnon N'F, Constantini C, Besansky NJ (2005). Rangewide population genetic structure of the African malaria vector *Anopheles funestus*. Mol Ecol.

[B12] Lehmann T, Licht M, Elissa N, Maega BTA, Chimumbwa JM, Watsenga FT, Wondji CS, Simard F, Hawley WA (2003). Population structure of *Anopheles gambiae *in Africa. J Heredity.

[B13] Coluzzi M, Sabatini A, Petrarca V, Di Deco MA (1979). Chromosomal differentiation and adaptation to human environments in the *Anopheles gambiae *complex. Trans R Soc Trop Med Hyg.

[B14] Wang R, Zheng L, Touré YT, Dandekar T, Kafatos FC (2001). When genetic distance matters: Measuring genetic differentiation at microsatellite loci in whole-genome scans of recent and incipient mosquito species. PNAS.

[B15] Forattini OP (1987). Exophilic behavior of *Anopheles darlingi *Root in a southern region of Brazil. Rev Saude Publica.

[B16] Hudson JE (1984). *Anopheles darlingi *Root (Diptera: Culicidae) in the Suriname rain forest. Bull Entomol Res.

[B17] Charlwood JD (1996). Biological variation in *Anopheles darlingi *Root. Mem Inst Oswaldo Cruz.

[B18] Quinones ML, Suarez MF (1990). Indoor resting heights of some anophelines in Colombia. J Am Mosq Control Assoc.

[B19] Faran ME, Linthicum KJ (1981). A handbook of Amazonian species of *Anopheles *(*Nyssorhynchus*). Mosq Syst.

[B20] Harbach RE, Roberts DR, Manguin S (1993). Variation in the hindtarsal markings of *Anopheles darlingi *(Diptera: Culicidae) in Belize. Mosq Syst.

[B21] Kreutzer RD, Kitzmiller JB, Ferreira E (1972). Inversion polymorphism in the salivary gland chromosomes of *Anopheles darlingi *Root. Mosq News.

[B22] Tadei WP, Santos JMM, Rabbani MG (1982). Biologia de anofelinos amazônicos. V. Polimorfismo cromossômico de *Anopheles darlingi *Root (Diptera: Culicidae). Acta Amazonica.

[B23] Steiner WWM, Narang S, Kitzmiller JB, Swofford DL, Steiner WWM, Tabachnick JW, Rai KS, Narang S (1982). Genetic divergence and evolution in neotropical *Anopheles *(Subgenus *Nyssorhynchus*). Recent Developments in the Genetics of Insect Disease Vectors.

[B24] Rosa-Freitas MG, Broomfield G, Priestman A, Milligan PJ, Momen H, Molyneux DH (1992). Cuticular hydrocarbons, isoenzymes and behavior of three populations of *Anopheles darlingi *from Brazil. J Am Mosq Control Assoc.

[B25] Dos Santos JM, Tadei WP, Contel EP (1996). Electrophoretic analysis of 11 enzymes in natural populations of *Anopheles *(*N*.) *darlingi *Root, 1926 (Diptera: Culicidae) in the Amazon region. Acta Amazonica.

[B26] Freitas-Sibajev MGR, Conn J, Mitchell SE, Cockburn AF, Seawright JA, Momen H (1995). Mitochondrial DNA and morphological analyses of *Anopheles darlingi *populations from Brazil (Diptera: Culicidae). Mosq Syst.

[B27] Conn JE, Rosa-Freitas MG, Luz SLB, Momen H (1999). Molecular population genetics of the primary Neotropical malaria vector *Anopheles darlingi *using mtDNA. J Am Mosq Control Assoc.

[B28] Mirabello L, Conn JE (2006). Molecular population genetics of the malaria vector *Anopheles darlingi *in Central and South America. Heredity.

[B29] Malafronte RS, Marrelli MT, Marinotti O (1999). Analysis of ITS2 DNA sequences from Brazilian *Anopheles darlingi *(Diptera: Culicidae). J Med Entomol.

[B30] Besansky NJ, Bedell JA, Benedict MQ, Mukabayire O, Hilfiker D, Collins FH (1995). Cloning and characterization of the *white *gene from *Anopheles gambiae*. Insect Mol Biol.

[B31] Krzywinski J, Besansky NJ (2002). Frequent intron loss in the *white *gene: a cautionary tale for phylogeneticists. Mol Biol Evol.

[B32] Harbach RE (2004). The classification of genus Anopheles (Diptera: Culicidae): a working hypothesis of phylogenetic relationships. Bull Entomol Res.

[B33] Coluzzi M, Sabatini A, Della Torre A, Dideco MA, Petrarca V (2002). A polytene chromosome analysis of the *Anopheles gambiae *species complex. Science.

[B34] della Torre A, Costantini C, Besansky NJ, Caccone A, Petrarca V, Powell JR, Coluzzi M (2002). Speciation within *Anopheles gambiae *– the glass is half full. Science.

[B35] Stump AD, Shoener JA, Costantini C, Sagnon N, Besansky NJ (2005). Sex-linked differentiation between incipient species of *Anopheles gambiae*. Genetics.

[B36] Powell JR, Petrarca V, della Torre A, Caccone A, Coluzzi M (1999). Population structure, speciation, and introgression in the *Anopheles gambiae *complex. Parassitologia.

[B37] Conn JE, Vineis JH, Bollback JP, Onyabe DY, Wilkerson RC, Póvoa MM (2006). Population structure of the malaria vector *Anopheles darlingi *in a malaria-endemic region of eastern Amazonian Brazil. Am J Trop Med Hyg.

[B38] Scarpassa VM, Conn JE (2007). Population genetic structure of the major malaria vector *Anopheles darlingi *(Diptera: Culicidae) from the Brazilian Amazon, using microsatellite markers. Mem Inst Oswaldo Cruz.

[B39] Walton C, Handley JM, Collins FH, Baimai V, Harbach RE, Deesin V, Butlin RK (2001). Genetic population structure and introgression in *Anopheles dirus *mosquitoes in South-east Asia. Mol Ecol.

[B40] Michel AP, Guelbeogo WM, Grushko O, Schemerhorn BJ, Kern M, Willard MB, Sagnon N'F, Costantini C, Besansky NJ (2005). Molecular differentiation between chromosomally defined incipient species of *Anopheles funestus*. Insect Mol Biol.

[B41] Michel AP, Grushko O, Guelbeogo WM, Lobo NF, Sagnon N'F, Costantini C, Besansky NJ (2006). Divergence with gene flow in *Anopheles funestus *from the Sudan savanna of Burkina Faso, West Africa. Genetics.

[B42] Pan American Health Organization (2006). 2006 Malaria in the Americas Data Tables.

[B43] Need J, Rogers E, Phillips I (1993). Mosquitoes (Diptera: Culicidae) captured in the Iquitos area of Peru. J Med Entomol.

[B44] Fernandez R, Carbajal F, Quintana J, Chauca H, Watts DM (1996). Presencia del *A. (N) darlingi *(Diptera: Culicidae), en alrededores de la ciudad de Iquitos, Loreto-Peru. Sociedad Peruana de Enfermedades Infecciosas y Tropicales.

[B45] Aramburu Guarda J, Ramal Asayag C, Witzig R (1999). Malaria reemergence in the Peruvian Amazon region. Emerg Infect Dis.

[B46] Schoeler GB, Flores-Mendoza C, Fernandez R, Davila JR, Zyzak M (2003). Geographical distribution of *Anopheles darlingi *in the Amazon Basin region of Peru. J Am Mosq Control Assoc.

[B47] Conn JE, Bollback J, Onyabe D, Robinson T, Wilkerson R, Póvoa M (2001). Isolation of polymorphic microsatellite markers from the malaria vector *Anopheles darlingi*. Mol Ecol Notes.

[B48] Deane LM, Causey OR, Deane MP (1946). An illustrated key by adult female characteristics for identification of thirty-five species of Anopheline from the northeast and Amazon regions of Brazil, with notes on the malaria vectors (Diptera: Culicidae). Am J Trop Med Hyg.

[B49] Goudet J (2002). FSTAT: A program to estimate and test gene diversities and fixation indices (version 2.9.3). http://www2.unil.ch/popgen/softwares/fstat.htm.

[B50] Brookfield JF (1996). A simple new method for estimating null allele frequency from heterozygote deficiency. Mol Ecol.

[B51] Van Oosterhout C, Hutchinson WF, Wills DPM, Shipley PF (2004). Micro-Checker: software for identifying and correcting genotyping errors in microsatellite data. Mol Ecol Notes.

[B52] Schneider S, Roessli D, Excoffier L (2000). Arlequin: A software for population genetic data.

[B53] Raymond M, Rousset F (1995). GENEPOP (version 1.2): population genetics software for exact tests and ecumenicism. J Heredity.

[B54] Slatkin M (1995). A measure of population subdivision based on microsatellite allele frequencies. Genetics.

[B55] Gaggiotti OE, Lange O, Rassmann K, Gliddon C (1999). A comparison of two indirect methods for estimating average levels of gene flow using microsatellite data. Mol Ecol.

[B56] Mantel N (1967). The detection of disease clustering and a generalized regression approach. Cancer Research.

[B57] Nei M, Tajima F, Tateno Y (1983). Accuracy of estimated phylogenetic trees from molecular data. II. Gene frequency data. J Mol Evol.

[B58] Kumar S, Tamura K, Nei M (2004). MEGA3: Integrated software for Molecular Evolutionary Genetics Analysis and sequence alignment. Briefings in Bioinformatics.

[B59] Pritchard JK, Stephens M, Donnelly P (2000). Inference of population structure using multilocus genotype data. Genetics.

[B60] Falush D, Stephens M, Pritchard JK (2003). Inference of population structure using multilocus genotype data: Linked loci and correlated allele frequencies. Genetics.

[B61] Cornuet JM, Luikart G (1996). Description and power analysis of two tests for detecting recent population bottlenecks from allele frequency data. Genetics.

[B62] Kimmel M, Chakraborty R, King JP, Bamshad M, Watkins WS, Jorde LB (1998). Signatures of population expansion in microsatellite repeat data. Genetics.

[B63] King JP, Kimmel M, Chakraborty R (2000). A power analysis of microsatellite-based statistics for inferring past populations growth. Mol Biol Evol.

[B64] Ellegren H (2000). Microsatellite mutations in the germline: implications for evolutionary inference. Trends Genet.

[B65] Donnelly MJ, Licht MC, Lehmann T (2001). Evidence for recent population expansion in the evolutionary history of the malaria vectors *Anopheles arabiensis *and *Anopheles gambiae*. Mol Biol Evol.

[B66] Peel D, Ovenden JR, Peel SL (2004). NeEstimator: software for estimating effective population size, Version 1.3.

[B67] Lounibos L, Conn JE (2000). Malaria vector heterogeneity in South America. Am Entomol.

[B68] Molina-Cruz A, de Merida AM, Mills K, Rodriguez F, Schoua C, Yurrita MM, Molina E, Palmieri M, Black IVWC (2004). Gene flow among *Anopheles albimanus *populations in Central America, South America, and the Caribbean assessed by microsatellites and mitochondrial DNA. Am J Trop Med Hyg.

[B69] Lehmann T, Besansky NJ, Hawley WA, Fahey TG, Kamau L, Collins FH (1997). Microgeographic structure of *Anopheles gambiae *in western Kenya based on mtDNA and microsatellite loci. Mol Ecol.

[B70] dos Santos JM, Maia J, de F, Tadei WP, Rodriques GA (2003). Isoenzymatic variability among five *Anopheles *species belonging to the *Nyssorhynchus *and *Anopheles *subgenera of the Amazon region, Brazil. Mem Inst Oswaldo Cruz.

[B71] dos Santos JMM, Lobo JA, Tadei WP, Contel EPB (1999). Intrapopulational genetic differentiation in *Anopheles *(*N*.) *darlingi *Root, 1926 (Diptera: Culicidae) in the Amazon region. Genet Mol Biol.

[B72] Donnelly MJ, Simard F, Lehmann T (2002). Evolutionary studies of malaria vectors. Trends Parasitol.

[B73] Wright S (1978). Evolution and Genetics of Populations Variability among and within Populations.

[B74] Carnahan J, Zheng L, Taylor CE, Toure YT, Norris DE, Dolo G, Diuk-Wasser M, Lanzaro GC (2002). Genetic differentiation of *Anopheles gambiae *s.s. populations in Mali, west Africa, using microsatellite loci. J Hered.

[B75] Lehmann T, Hawley WA, Grebert H, Danga M, Atieli F, Collins FH (1999). The Rift Valley complex as a barrier to gene flow for *Anopheles gambiae *in Kenya. J Hered.

[B76] Pinedo-Cancino V, Sheen P, Tarazona-Santos E, Oswald WE, Jeri C, Vittor AY, Patz JA, Gilman RH (2006). Limited diversity of *Anopheles darlingi *in the Peruvian Amazon region of Iquitos. Am J Trop Med Hyg.

[B77] Slatkin M (1985). Rare alleles as indicators of gene flow. Evolution.

[B78] Lanzaro GC, Touré YT, Carnahan J, Zheng L, Dolo G, Traoré S, Petrarca V, Vernick KD, Taylor CE (1998). Complexities in the genetic structure of *Anopheles gambiae *populations in west Africa as revealed by microsatellite DNA analysis. PNAS.

[B79] Whitlock MC, McCauley DE (1999). Indirect measures of gene flow and migration: FST not equal to 1/(4Nm +1). Heredity.

[B80] Cohuet A, Dia I, Simard F, Raymond M, Rousset F, Antonio-Nkondjio C, Awono-Ambene PH, Wondji CS, Fontenille D (2005). Gene flow between chromosomal forms of the malaria vector *Anopheles funestus *in Cameroon, Central Africa, and its relevance in malaria fighting. Genetics.

