# Supplementary material for: Microsatellite data suggest significant population structure and differentiation within the malaria vector Anopheles darlingi in Central and South America
Source: BMC Ecol. 2008 Mar 26;8:3. doi: 10.1186/1472-6785-8-3 (PMC2292152; doi:10.1186/1472-6785-8-3)
Supplement: Additional File 1 — Summary of microsatellite variation at 5–8 loci for A. darlingi in Central America, Peru and Brazil. The data provided represent the number of alleles, heterozygosity and inbreeding coeffient values of each microsatellite locus at each locality. [file 1472-6785-8-3-S1.doc]

Additional File 1. Summary of microsatellite variation at 5-8 loci for *A. darlingi* in Central America, Peru and Brazil.

Peru^1^ Brazil^1^ .. Central America^2^ .

NAU PRT MAZ SHP CAT PCO ZUN BV PLT CAV GOL SIB SPB ELP SRO

Locus *N*=53 *N*=35 *N*=50 *N*=58 *N*=50 *N*=52  *N*=52 *N*=57 *N*=58 *N*=45 *N*=41 *N*=47 *N*=47 *N*=47 *N*=49

*ADC01*

*A* 15 14 16 19 20 18 14 19 27 -- -- -- -- -- --

*R*_S_ 13.06 13.97 14.77 17.45 18.59 15.95 13.78 11.70 15.84 -- -- -- -- -- --

*H*_O_ 0.825 0.926 0.907 0.936 0.928 0.916 0.912 0.894 0.911 -- -- -- -- -- --

*F*_IS_ 0.062 0.075 0.118 0.005 0.034 0.034 0.051 0.022 0.122 -- -- -- -- -- --

*ADC02*

*A* 5 5 5 8 11 6 5 7 22 7 5 6 6 6 6

*R*_S_ 4.47 5.00 4.36 7.70 9.68 5.41 4.88 6.24 14.14 6.79 4.86 5.95 5.86 6.00 5.92

*H*_O_ 0.439 0.557 0.497 0.716 0.739 0.456 0.509 0.739 0.933 0.679 0.537 0.577 0.471 0.512 0.419

*F*_IS_ 0.269 -0.232 0.156 0.422* 0.351* 0.113 0.244 -0.028 0.339* 0.006 0.000 0.316 -0.011 0.023 0.205

*ADC28*

*A* 8 8 8 7 9 7 10 6 11 3 3 4 4 5 4

*R*_S_ 7.94 8.00 7.87 6.49 8.48 6.99 9.61 5.74 8.13 2.96 3.00 3.70 3.95 4.89 3.74

*H*_O_ 0.820 0.816 0.800 0.718 0.738 0.785 0.839 0.749 0.827 0.147 0.224 0.123 0.221 0.228 0.177

*F*_IS_ 0.125 -0.016 0.175 -0.081 0.025 0.167 0.106 -0.049 0.244 -0.055 -0.090 -0.036 -0.081 0.122 -0.061

*ADC29*

*A* 9 10 11 11 11 9 10 14 28 -- -- -- -- -- --

*R*_S_ 8.22 9.97 9.61 10.52 10.22 7.87 9.06 10.99 16.82 -- -- -- -- -- --

*H*_O_ 0.705 0.596 0.643 0.835 0.776 0.540 0.663 0.881 0.946 -- -- -- -- -- --

*F*_IS_ 0.277* 0.137 0.253 0.298* 0.201 0.217 0.275 0.291* 0.511* -- -- -- -- -- --

*ADC110*

*A* 8 6 6 8 9 6 6 10 12 5 5 5 6 5 5

*R*_S_ 7.26 6.00 6.00 7.39 8.25 5.99 5.65 7.47 9.47 4.97 4.88 4.89 5.92 4.92 4.94

*H*_O_ 0.743 0.769 0.760 0.805 0.791 0.788 0.665 0.797 0.883 0.613 0.629 0.469 0.645 0.546 0.572

*F*_IS_ 0.010 -0.148 0.105 0.079 -0.062 -0.050 -0.071 0.032 0.284* 0.279 0.149 0.360 0.444* 0.530* 0.272

*ADC137*

*A* 9 9 10 8 9 8 9 12 14 6 4 6 4 3 4

*R*_S_ 8.95 8.97 9.88 7.37 8.68 7.99 8.27 8.88 10.40 5.97 3.88 5.89 3.86 3.00 3.75

*H*_O_ 0.853 0.843 0.833 0.784 0.863 0.861 0.820 0.857 0.884 0.652 0.568 0.568 0.469 0.425 0.490

*F*_IS_ 0.049 0.322* 0.100 0.172 0.252* 0.151 0.156 -0.062 0.151 0.037 -0.116 0.073 0.035 0.047 0.022

*ADC138*

*A* 6 7 9 8 9 7 7 8 18 2 3 2 5 2 2

*R*_S_ 5.89 6.97 8.01 7.65 8.54 6.27 6.72 6.28 12.75 2.00 2.90 2.00 4.84 2.00 2.00

*H*_O_ 0.493 0.645 0.633 0.774 0.692 0.630 0.576 0.785 0.904 0.476 0.398 0.416 0.520 0.441 0.487

*F*_IS_ 0.159 0.026 0.273 0.207 0.162 0.268 0.131 0.227 0.425* 0.516 -0.004 0.675* 0.556* 0.447 0.362

*ADC107*

*A*  -- -- -- -- -- -- -- 9 14 -- -- -- -- -- --

*R*_S_ -- -- -- -- -- -- -- 6.59 11.18 -- -- -- -- -- --

*H*_O_ -- -- -- -- -- -- -- 0.489 0.882 -- -- -- -- -- --

*F*_IS_ -- -- -- -- -- -- -- 0.257 0.345* -- -- -- -- -- --

Based on 7-8 loci:

Mean *R*_S_ 7.97 8.41 8.64 9.22 10.35 8.07 8.28 7.99 12.34 -- -- -- -- -- --Mean *H*_O_ 0.697 0.736 0.725 0.795 0.790 0.711 0.712 0.774 0.896 -- -- -- -- -- --Based on 5 loci:

Mean *R*_S_ 6.47 6.69 6.74 6.75 7.89 6.13 6.55 7.53 12.53 4.54 3.90 4.49 4.89 4.16 4.07

Mean *H*_O_ 0.669 0.726 0.705 0.759 0.765 0.704 0.682 0.785 0.886 0.513 0.471 0.431 0.465 0.430 0.429

^1^, genotype 1 localities; ^2^, genotype 2 localities; --, no data; *N*, sample size; *A*, number of alleles; *R*_S_, allelic richness; *H*_O_, observed heterozygosity; *F*_IS_, inbreeding coefficient; *, indicates a significant value after Bonferroni correction.
